# Association between hospital competition and quality of prostate cancer care

**DOI:** 10.1186/s12913-023-09851-4

**Published:** 2023-08-05

**Authors:** Ravishankar Jayadevappa, S. Bruce Malkowicz, Neha Vapiwala, Thomas J. Guzzo, Sumedha Chhatre

**Affiliations:** 1 Department of Medicine, Perelman School of Medicine, University of Pennsylvania, Philadelphia, PA US; 2 Department of Surgery, Division of Urology, Perelaman School of Medicine, University of Pennsylvania, Philadelphia, PA US; 3 Abramson Cancer Center, University of Pennsylvania, Philadelphia, PA US; 4 Leonard Davis Institute of Health Economics, University of Pennsylvania, Philadelphia, PA US; 5 Department of Radiation Oncology, Perelman School of Medicine, University of Pennsylvania, Philadelphia, US; 6 Department of Psychiatry, Perelman School of Medicine, University of Pennsylvania, Philadelphia, PA US; 7 Corporal Michael J. Crescenz VAMC, Philadelphia, PA US

**Keywords:** Hospital competition, SEER-Medicare, Localized prostate cancer, Older adults, Outcomes of care

## Abstract

**Background:**

Hospitals account for approximately 6% of United States’ gross domestic product. We examined the association between hospital competition and outcomes in elderly with localized prostate cancer (PCa). We also assessed if race moderated this association.

**Methods:**

Retrospective study using Surveillance, Epidemiology, and End Results (SEER) – Medicare database. Cohort included fee-for-service, African American and white men aged ≥ 66, diagnosed with localized PCa between 1998 and 2011 and their claims between 1997 and 2016.

We used Hirschman-Herfindahl index (HHI) to measure of hospital competition. Outcomes were emergency room (ER) visits, hospitalizations, Medicare expenditure and mortality assessed in acute survivorship phase (two years post-PCa diagnosis), and long-term mortality. We used Generalized Linear Models for analyzing expenditure, Poisson models for ER visits and hospitalizations, and Cox models for mortality. We used propensity score to minimize bias.

**Results:**

Among 253,176 patients, percent change in incident rate of ER visit was 17% higher for one unit increase in HHI (IRR: 1.17, 95% CI: 1.15–1.19). Incident rate of ER was 24% higher for whites and 48% higher for African Americans. For one unit increase in HHI, hazard of short-term all-cause mortality was 7% higher for whites and 11% lower for African Americans. The hazard of long-term all-cause mortality was 10% higher for whites and 13% higher for African Americans.

**Conclusions:**

Lower hospital competition was associated with impaired outcomes of localized PCa care. Magnitude of impairment was higher for African Americans, compared to whites. Future research will explore process through which competition affects outcomes and racial disparity.

**Supplementary Information:**

The online version contains supplementary material available at 10.1186/s12913-023-09851-4.

## Introduction

Hospitals are an important sector of the United States’ (US) economy and account for approximately 6% of its gross domestic product [1]. Hospital Competition Act of 2019 stresses the importance of hospital competition in improving quality of health services and outcomes [2].

The move towards consolidation of the US healthcare markers has resulted in a complex relationship between hospital competition, access to care, quality of care and outcomes. Hospital competition affects a patient’s quality of care and outcomes in many ways given the differences in patient demographics, their preferences and decision-making [3]. For stakeholders such as healthcare providers, decision-makers and purchasers, assessing the quality of health services and outcomes is important to satisfy the demand for quality transparency, cost control and lowering the variations in clinical practice.

Impact of competition on quality of care and outcomes can go either way: competition may forces hospitals to cut prices and quality, or to improve quality [3–7]. Less competitive areas contain less densely populated areas [8]. Hospitals in these areas tend to be non-teaching hospitals, are often smaller and so benefit less from scale economies and have less investment in high tech equipment [8]. If competitive forces remain low, such hospitals have no incentives to take more complex and costly patients. If less-costly patients are also healthy with fewer or no comorbidities, then there will be a negative association between quality of care, outcomes and competition. This is not attributable to the relative strength of quality signals but is the outcome of selection that can accompany competition. Thus, hospital competition can play an important role in the quality of care and outcomes of care.

Hospital competition can have socially beneficial as well as harmful effects. Hospital competition can have a major influence on the configuration of cancer services [3], cost [9], and hospital admissions [10]. Prior studies have reported mixed results regarding the association between competition and quality [3–6, 11, 12]. Studies have demonstrated that when government regulation “fixes” prices, and thus forces providers to compete on quality, it can result in higher-quality care and better outcomes [13–15]. At the same time government price regulations and other policies such as barriers to entry, may act as incentives to consolidate [14, 15].

Prostate cancer (PCa) is the most frequent cancer among men in the US and exerts substantial burden on the healthcare system. Hospital and physician characteristics, particularly volume, are associated with the variations in PCa outcomes including cost, health service use, complications, and mortality. Hospital volume is often considered as a surrogate for quality of care, and studies have suggested that referring patients from low-volume to high-volume provider may improve the quality of care and reduce health service use and cost [16–18]. Among surgically treated patients, those treated by high-volume surgeons had half the risk of complications and shorter length of stay compared to those treated by low-volume surgeons [16, 17, 19]. Volume based arguments indicate a significant gain in the overall quality of care if one redirects complicated surgery from low to high volume physicians or hospitals [16, 17, 19].

As US hospital market is becoming more consolidated, it is important to assess how hospital competition can play a role in improving health care quality and outcomes and reduce cost [20]. Unlike hospital volume, association of hospital competition with health service use, expenditure and mortality in the context of PCa care remains unclear. Additionally, impact of the interaction of competition and race on outcomes of PCa care is yet to be explored. Our study is based on the Donabedian model of quality of care which consists of three components: structure, process and outcomes of care [21–24]. Per this model we have assessed the association between structure (hospitals) and outcomes of care. Thus, in this paper, we examined the association between hospital competition with health service use, Medicare expenditure, all-cause mortality and PCa-specific mortality, in acute survivorship phase (two years post-PCa diagnosis), and long-term mortality in older Medicare fee-for-service beneficiaries with localized PCa. We also assessed if these associations varied by race (African American and white).

## Methods

### Data and cohort selection

We used Surveillance, Epidemiology, and End Results (SEER)-Medicare database of the National Cancer Institute for the period between 1998 and 2016. These data provide information about Medicare beneficiaries with cancer who reside in the SEER regions. The SEER program collects data on cancer incidence, treatment, and mortality from sixteen SEER sites and encompasses 26% of the US population [25]. We used following SEER-Medicare files: (1) Patient Entitlement and Diagnosis File (PEDSF) containing SEER registry data and Medicare entitlement information; (2) Medicare Provider Analysis and Review file (MEDPAR) containing claims for hospital inpatient and skilled nursing facility stays; (3) Outpatient Standard Analytic File (outpatient) containing claims for hospital outpatient services; and (4) Physician/Supplier File (NCH) containing claims for physician/other medical services. We also used hospital file from SEER-Medicare and from the American Hospital Association (AHA) [26]. This paper follows the strengthening the Reporting of Observational Studies in Epidemiology (STROBE) reporting guidelines. The local institutional review board approved this study.

### Study cohort

Our study cohort consisted of African American and white male fee-for-service Medicare enrollees who were diagnosed with localized PCa between 1998 and 2011 and were aged 66 or older at the time of diagnosis. Inpatient, outpatient and provider service claims for this cohort between 1997 and 2016 were used.

#### Acute survivorship phase

Majority of the PCa patients receive treatment during the two-year period following PCa diagnosis, and may continue to experience treatment related morbidity and mortality beyond this phase. Therefore, we operationalized acute survivorship phase as the two-year period following PCa diagnosis [27].

### Dependent variables – outcomes of care

Short-term outcomes that were assessed over the acute survivorship phase were emergency room (ER) visits, any hospitalizations, Medicare expenditure (Medicare reimbursement) and mortality (all-cause and PCa-specific). We also assessed long-term mortality (all-cause and PCa-specific) over the follow-up of up to 20 years (i.e., up to 12/31/2016).

#### ER visits

We used outpatient claims to determine ER visits that did not result in hospitalizations (Revenue Center Codes 0450–0459, 0981).

#### Hospitalizations

We used MEDPAR files of SEER-Medicare to identify all hospitalizations.

#### Medicare expenditure

Medicare expenditure was the sum of Medicare reimbursements for all hospitalizations, outpatient and provider services in the acute survivorship phase.

#### Mortality

The PEDSF consists of mortality data reported by both SEER and Medicare. SEER only reports month and year of death, therefore, we assigned middle of the month as the day of death to construct the SEER date of death. Medicare date of death was constructed using the Medicare day, month, and year of death. A patient was coded as deceased if SEER and/or Medicare date of death was available. Those who were alive at the end of study period (12/31/2016) were censored. We extracted SEER reported prostate cancer-specific mortality data from PEDSF.

### Independent variables

#### Hospital competition

The Hirschman-Herfindahl index (HHI) is a common measure of competition and market competitiveness (HHI) [28, 29]. The HHI is measured at the hospital level and is the sum of squares of market share in a particular Hospital Service Area (HSA) and can range from near zero to 10,000.

$$\mathrm{HHI}={\sum }_{i=1}^{n}{S}_{i}^{2}$$

A market for PCa patients will have an HHI near zero if there were several competing hospitals from which patients can receive care [15, 30]. HSAs represent local healthcare market and were defined by assigning ZIP codes to the hospital area where the greatest proportion of their Medicare residents were hospitalized, leading to 3,436 HSAs [30].

### Hirschman—Herfindahl index (HHI) calculation

The HHI was calculated as the ratio of hospital count over sum of hospital counts for a given HSA. Specific steps in the calculation of HHI are as following: (1) Identify all hospitals from hospitalizations and outpatient services for each patient during the acute survivorship phase. (2) Create a frequency count for each hospital. (3) Use American Hospital Association’s annual HSA-hospital crosswalk to create a master file of hospitals and HSA. (4) Cross the files created in steps (2) and (3) to attach a HSA to each hospital from (2). (5) Sum the hospital count for each HSA. (6) Create a proportion where numerator is the hospital count and denominator is the sum of hospital count by HSA. (7) Square the proportion from step 6 and sum it over a HSA. Result is the HHI for a given HSA. The HHI ranges from 0 to 10,000 [20], lower value indicates higher competition.

### Treatment type

Exclusive categories of treatment received during acute survivorship phase were surgery (alone or with radiation, chemotherapy, and/or hormone therapy), radiation therapy/chemotherapy/hormone therapy (alone or combinations of), and no treatment. Treatment was extracted from both PEDSF and Medicare claims (see eTable 1 for ICD codes and CPT codes for treatment).

### Demographic and clinical characteristics

Data on demographic variables (age at diagnosis, marital status, race (African American or white), and census tract poverty index), were obtained from PEDSF. Clinical attributes were co-morbidity measured as Charlson comorbidity index score, and cancer grade. Data on cancer grade was obtained from PEDSF. We used Medicare inpatient, outpatient and provider claims from the one-year before PCa diagnosis to develop Charlson co-morbidity index [31]. Localized PCa cases were identified by selecting ‘localized’ codes for the SEER variable ‘Summary stage 2000’ from PEDSF. This variable is derived from Collaborative Stages (CS) for 2004 + and extent of disease (EOD) prior to that, and is used in most SEER publications [32].

### Statistical analysis

All analyses were conducted at the PCa patient level. The main exposure variable, hospital competition, was operationalized as HHI score (continuous variable). First, we used multinomial logistic regression to examine the association between hospital competition and PCa treatment. Short-term outcomes (assessed over the acute survivorship phase) were ER visits, any hospitalizations, Medicare expenditure and mortality (all-cause and PCa specific). For ER visits and hospitalizations, we used Poisson regression models. For Medicare expenditure, we used generalized linear models (GLM) with log-link (gamma distribution). Cox proportional hazard model was used for all-cause mortality and Fine and Gray model of competing risk was used for PCa-specific mortality. Long-term outcome was mortality (all-cause and PCa specific) over the follow-up of up to 20 years (as of 12/31/2016). We ran two set of models for each outcome. First set yielded the main effects of hospital competition. The second set included interaction between race and hospital competition. For Poisson models, we reported incidence rate ratios (IRR) and 95% confidence interval (CI). For GLM models we reported the exponentiated beta estimates (e^β^) and 95% CIs, and for the survival models, we reported hazard ratio (HR) and 95%CI.

Treatment assignment for PCa is non-random, therefore, we used propensity score to address selection bias [33]. Using multi-nominal logistic regression, we estimated for each patient the probability (propensity) of receiving a specific PCa treatment after accounting for age, race and ethnicity, marital status, cancer grade, census tract poverty indicator, and Charlson co-morbidity score. All analytical models were weighted by the inverse of propensity score. Statistical Analysis System (SAS), Version 9.4 (SAS Institute, Cary, NC, USA) was used for analysis.

## Results

**Fig. 1:**
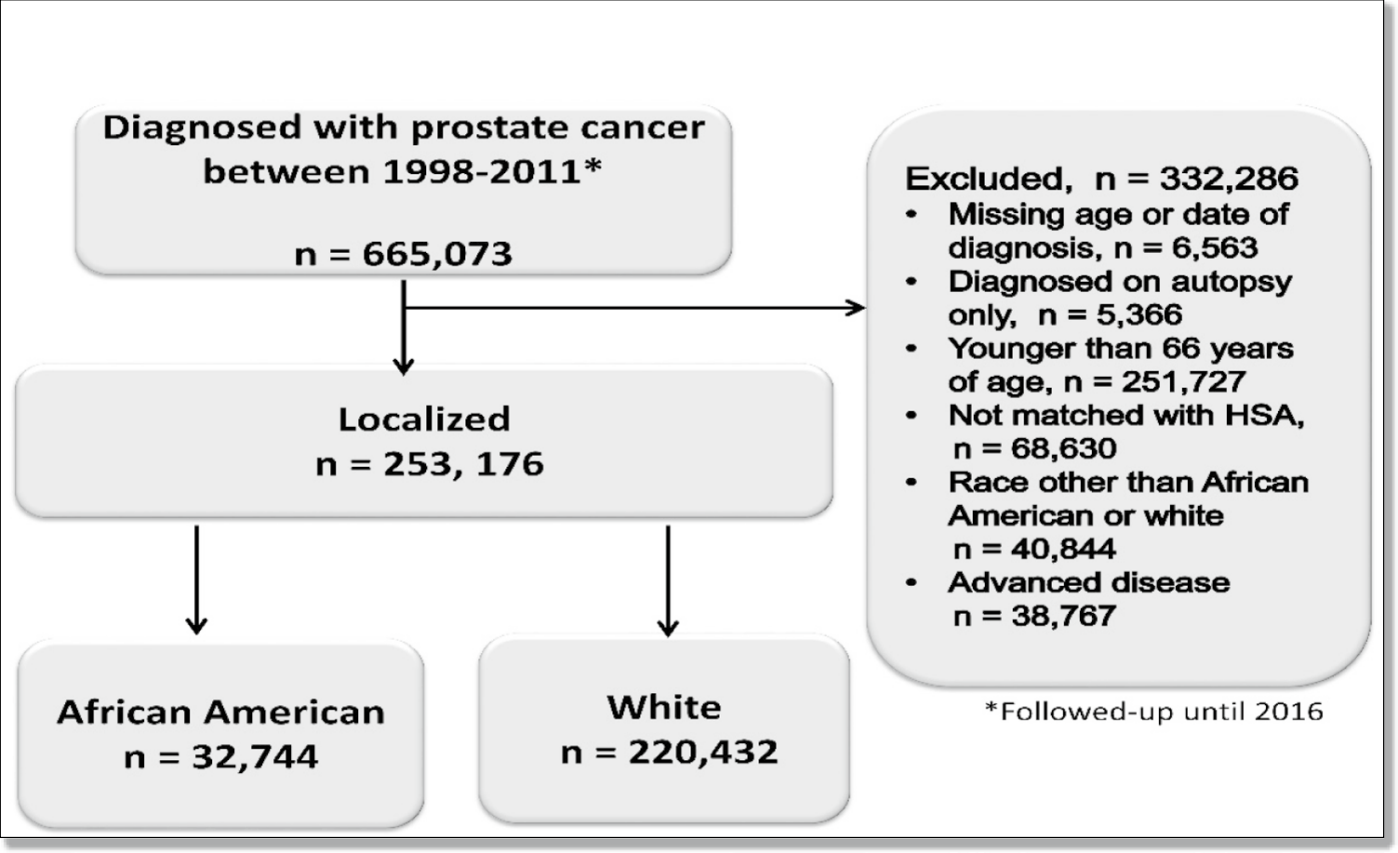
Cohort selection for prostate cancer cases diagnosed between 1998 and 2011 The mean annual HHI score between 1998 and 2011 remained mostly stable (eFigure 1). In Table 1, we show the comparison of demographic and clinical characteristics between four quartiles of HHI score

Our cohort consisted of 253,176 Medicare fee-for-service patients with localized PCa. Of these 32,744 were African American and 220,432 were white (see Fig. 1 for flow chart) Table 1.

**Table 1:** Comparison of socio-demographic and clinical characteristics for localized prostate cancer patients by race, by quartiles of HHI score, *n* = 253, 176

	**Quartile 1 *N* = 62,483**	**Quartile 2 *N* = 64,189**	**Quartile 3 *N* = 60,291**	**Quartile 4 *N* = 66,213**
Race, n (%)^c^				
African American	1,305 (20.9)	7,605 (11.9)	7,034 (11.7)	5,020 (7.6.)
White	49,398 (79.1)	56,584 (88.2)	53,257 (88.3)	61,193 (92.4)
Age in years (mean ± std)^b^	74.2 ± 5.9	74.8 ± 6.1	74.9 ± 6.0	75.3 ± 6.2
Married, n (%)^c^	40,595 (64.9)	43,032 (67.0)	39,954 (66.3)	43,129 (65.1)
Census poverty index, n (%)^c^				
0%- < 5% poverty	16,874 (27.0)	18,569 (28.9)	20,508 (34.0)	20,432 (30.9)
5% to < 10% poverty	17,017 (27.2)	17,602 (27.4)	10,888 (28.0)	18,855 (28.5)
10% to < 20% poverty	15,683 (25.1)	17,165 (26.7)	14,522 (24.1)	17,402 (26.2)
0% to 100% poverty	12,731 (20.4)	10,539 (16.4)	8,151 (13.5)	9,193 (13.9)
Unknown	178 (0.28)	314 (0.49)	222 (0.37)	331 (0.50)
Comorbidity, n (%)^c^				
0	32,091 (51.4)	33,684 (52.5)	32,415 (55.8)	36,345 (54.9)
1–2	27,205 (43.5)	27,174 (42.3)	24,543 (40.7)	26,2375 (39.6)
≥ 3	3,187 (5.1)	3,331 (5.2)	3,333 (5.5)	3,613 (5.5)
Grade, n (%)^c^				
Well differentiated	1,279 (2.1)	1,612 (2.5)	1,369 (2.3)	1,832 (2.8)
Moderately differentiated	33,364 (53.4)	33,624 (52.4)	32,625 (54.1)	35,155 (53.1)
Poorly/Undifferentiated/	24,821 (39.7)	25,485 (39.7)	23,142 (38.4)	25,312 (38.2)
Unknown	3,019 (4.8)	3,468 (5.4)	3,155 (5.2)	3,914 (5.9)
Treatment, n (%)^c^				
Surgery	11,870 (19.0)	11,857 (18.5)	9,261 (15.4)	9,672 (14.6)
Radiation/Hormone	32,706 (52.3)	32,992 (51.4)	34,390 (57.0)	37,083 (56.0)
/Chemotherapy				
No treatment	17,907 (28.7)	19,340 (30.1)	16,640 (27.6)	19,452 (29.4)

^a^*P* for comparison of white and African American prostate cancer patients, with *P* < .05 denoting statistical significance

^b^T tests for comparison of means

^c^Chi sq test for comparison of proportions

Unadjusted comparison of outcomes between African Americans and white patients is presented in Table 2.

**Table 2:** Unadjusted comparison of health service use, Medicare expenditure and mortality outcomes in the follow-up period for localized prostate cancer patients by race, by quartiles of HHI score, *n* = 253, 176

	**Quartile 1 *N* = 62,483**	**Quartile 2 *N* = 64,189**	**Quartile 3 *N* = 60,291**	**Quartile 4 *N* = 66,213**
*Short term outcomes (acute survivorship phase i.e. two years post prostate cancer diagnosis)*				
ER Visits, n (%)^b^				
0	45,913 (73.5)	45,171 (70.5)	42,107 (69.8)	44,629 (67.4)
1–3	12,986 (20.8)	14,737 (22.9)	14,275 (23.7)	16,705 (25.2)
≥ 4	3,584 (5.7)	4,281 (6.7)	3,909 (6.5)	4,879 (7.4)
Hospitalizations, n (%)^b^				
0	32,079 (51.3)	32,154 (50.1)	32,465 (53.9)	35,601 (53.8)
1–3	26,534 (42.5)	27,543 (42.9)	23,695 (39.3)	25,906 (39.1)
≥ 4	3,870 (6.2)	4,492 (7.0)	4,131 (6.9)	4,706 (7.1)
All-cause mortality, n (%)^b^	5,073 (8.1)	5,678 (8.9)	5,162 (8.6)	6,171 (9.3)
Prostate cancer-specific mortality, n (%)^b^	975 (1.6)	1,062 (1.7)	904 (1.5)	1,272 (1.0)
Medicare expenditure ($),	22,827	22,972	25,607	24,392
mean ± SD	± 31,481	± 28,814	± 29,804	± 27,914
*Long term outcomes (up to 19 years post prostate cancer diagnosis)*				
All-cause mortality, n (%)^b^	34,218 (54.8)	37,765 (58.8)	35,040 (58.1)	40,048 (60.5)
Prostate cancer-specific mortality, n (%)^b^	4,880 (7.8)	5,367 (8.4)	4,747 (7.9)	5,837 (8.8)

^a^*P* for comparison of white and African American prostate cancer patients, with *P* < .05 denoting statistical significance

^b^Chi sq test for comparison of proportions

Multinomial logit model results showed that a one unit increase in HHI score was associated with 0.80 decrease in the relative log odds of receiving surgery vs. no treatment. Additionally, a one unit increase in HHI score was associated with 1.27 increase in the relative log odds of receiving radiation vs. no treatment, holding other covariates constant. Thus, lower competition (one unit increase in HHI score) was associated with lower likelihood of receiving surgery, and higher likelihood of receiving radiation, instead of no treatment (data not shown).

### Emergency room visits

As seen from Model 1 (Table 3), the percent change in the incident rate of ER visit was 17% higher for one unit increase in HHI score, holding other variables constant (IRR: 1.17, 95% CI: 1.15–1.19).

**Table 3:** Summary of 2 Series of Models on the interactive effects of race and hospital completion on ER visits, hospitalizations, Medicare expenditure, all-cause mortality and cancer-specific mortality,^a^ for localized prostate cancer patients, *n* = 253,176

	**Model 1: Main Effects**	**Model 2: Model 1 Plus Interaction**
***Short-term outcomes***		
** ER visit**	**IRR (95% CI)**^**b**^	**IRR (95% CI)**^**b**^
Race (African American)	1.16 (1.14, 1.18)	1.05 (1.02, 1.07)
HHI score	1.17 (1.15, 1.19)	1.24 (1.22, 1.27)
HHI x African American		1.19 (1.15, 1.23)
**Hospitalization**	**IRR (95% CI)**^**b**^	**IRR (95% CI)**^**b**^
Race (African American)	1.09 (1.08, 1.11)	1.08 (1.05, 1.10)
HHI score	1.03 (1.01, 1.09)	1.00 (0.98, 1.02)
HHI x African American		1.03 (1.00, 1.07)
**Medicare expenditure**	**e**^**β**^** (95% CI) **^**c**^	**e**^**β**^** (95% CI)**^**c**^
Race (African American)	1.01 (0.98, 1.02)	1.02 (0.98, 1.06)
HHI score	1.08 (1.05, 1.10)	1.06 (1.03, 1.09)
HHI x African American		0.96 (0.91, 1.02)
**All-cause Mortality**	**HR (95% CI)**^**d**^	**HR (95% CI)**^**d**^
Race (African American)	1.05 (1.01, 1.09)	1.23 (1.14, 1.32)
HHI score	1.03 (0.99, 1.07)	1.07 (1.03, 1.11)
HHI x African American		0.83 (0.75, 0.91)
**Prostate Cancer-specific Mortality**	**HR (95% CI)**^**d**^	**HR (95% CI)**^**d**^
Race (African American)	1.30 (1.21, 1.41)	1.61 (1.39, 1.89)
HHI score	0.95 (0.87, 1.04)	1.02 (0.92, 1.13)
HHI x African American		0.71 (0.57, 0.88)
***Long-term outcomes***		
**All-cause Mortality**	**HR (95% CI)**^**d**^	**HR (95% CI)**^**d**^
Race (African American)	1.05 (1.03, 1.06)	1.09 (1.06, 1.12)
HHI score	1.09 (1.08, 1.11)	1.10 (1.08, 1.12)
HHI x African American		1.03 (1.01, 1.08)
**Prostate Cancer-specific Mortality**	**HR (95% CI)**^**d**^	**HR (95% CI)**^**d**^
Race (African American)	1.27 (1.26, 1.32)	1.32 (1.23, 1.43)
HHI score	1.05 (1.01, 1.09)	1.07 (1.02, 1.12)
HHI x African American		0.99 (0.89, 1.10)

^a^Weighted by propensity score. All models were also adjusted for age, marital status, Charlson comorbidity score, grade and treatment

^b^*IRR* Incidence rate ratio

^c^*e*^*β*^ exponent of beta estimate; ^d^ *HR* Hazard ratio

Thus, lower competition was associated with higher ER visits. Model 2 showed statistically significant interaction between race and HHI score. For white PCa patients, the incident rate of ER visits was 24% higher (IRR:1.24, 95% CI: 1.22, 1.27) for one unit increase in HHI score. The effect of higher HHI score for African American PCa patient was 1.19 times that for whites (IRR = 1.19, 95% CI = 1.15, 1.23). The interaction effect represents by how much the effect of hospital competition differs between African American and white patients in multiplicative terms. Thus, the incident rate of ER visits was 48% higher for African Americans.

### Hospitalizations

Main effects of hospital competition (HHI score) for hospitalizations was significant (IRR: 1.03, 95%CI: 1.01–1.09). Model 2 showed statistically significant interaction between race and HHI score. For whites, the association between HHI and hospitalizations was not significant. The effect of higher HHI score for African Americans was 1.03 times that of their white counterparts (IRR = 1.03, 95% CI = 1.00, 1.07). Thus, the incident rate of hospitalizations was 3% higher for African Americans.

### Medicare expenditure

One unit increase in HHI score was associated with 8% increase in expenditure (e^β^: = 1.08, 95% CI, 1.05–1.10). However, the interaction between race and hospital competition was statistically non-significant.

### Short-term all-cause mortality

Main effects of hospital competition for short-term all-cause mortality was not significant (Hazard Ratio (HR): 1.03, 95% CI: 0.99, 1.07; Model 1). Interaction between race and hospital competition was significant. For white PCa patients, the hazard of all-cause mortality was 7% higher (HR: 1.07, 95% CI: 1.03, 1.11) for one unit increase in HHI score. The effect of higher HHI score for African American PCa patient was 0.83 times that for whites (HR: 0.83, 95% CI: 0.75 0.91). Thus, the hazard of short-term all-cause mortality was 11% lower for African Americans.

### Short-term PCa-specific mortality

Main effects of hospital competition (HHI score) for PCa-specific mortality was not statistically significant. Model 2 showed statistically significant interaction between race and hospital competition. The effect of higher HHI score for African American PCa patient was 0.71 times that of their white counterparts (HR: 0.71, 95% CI: 0.57, 0.88). Thus, the hazard of PCa specific mortality was 28% lower for African Americans.

### Long-term all-cause mortality

Main effects of hospital competition for long-term all-cause mortality (HR: 1.09, 95% CI: 1.08, 1.11) was observed (Model 1). One unit increase in HHI score (i.e., decrease in competition) was associated with 9% increase in hazard of long-term mortality. Interaction between race and competition was significant (Model 2). For white PCa patients, the hazard of long-term all-cause mortality was 10% higher (HR: 1.10, 95% CI: 1.08, 1.12) for one unit increase in HHI score. The effect of higher HHI score for African American PCa patient was 1.03 times that for whites (HR: 1.03, 95% CI: 1.01, 1.08). Thus, the hazard of long-term all-cause mortality was 13% higher for African Americans.

### Long-term PCa-specific mortality

The main effects of hospital competition for long-term PCa-specific mortality showed that for one unit increase in HHI score (i.e., lower competition), the hazard of PCa-specific mortality was 5% higher (HR: 1.05, 95% CI: 1.01, 1.09). However, interaction between race and hospital competition was not significant.

## Discussion

Hospital competition is an instrument for organizing the decisions about efficient use of resources [4, 33]. Hospital Competition Act of 2019 stresses the importance of hospital competition in improving the efficiency and quality of healthcare[1]. In our first of its kind study, we observed that lower competition was associated with impaired outcomes of ER visits, hospitalizations, Medicare expenditure and mortality. In our study, majority of the hospitals (more than 90%) served Medicare and Medicaid patients, two-thirds were non-profit, 55% were urban hospitals, and 28% were teaching hospital. Lower competition was associated with higher use of health services, and Medicare expenditure. The magnitude of the association between lower competition and higher ER use differed by race. The association between hospital competition and Medicare expenditure did not differ by race. Short-term mortality (all-cause and PCa-specific) was not associated with hospital competition. Lower competition was associated with higher hazard of long-term mortality (all-cause mortality and PCa-specific). The association between low competition and long-term all-cause mortality differed by race, and the hazard was higher for African Americans than that for whites.

There exists a debate about the role of hospital competition in healthcare quality and cost [34–36]. Policies have been introduced for improving the quality of care and outcomes by stimulating competition between hospitals and allowing hospital choice [37–40]. When hospitals compete on quality and not on price, this is expected to attract patients [13, 38–40]. In a UK based study, PCa patients receiving radical prostatectomy in a highly competitive environment reported lower urinary complications, ER admissions within 30 days, and shorter length of stay, irrespective of hospital volume [41].

In accordance with the economic theory, our study suggests that increased competition can lead to lower prostate cancer care costs. Less competition among practices was associated with substantially higher prices paid for office visits to physicians [9]. Competitive environment affects the quality of care delivered by hospitals, which, in turn, affects outcomes, and quality and length of life. Hospital volume and its effect on treatment, quality of care and other outcomes have been explored [16, 17, 19]. Hospital volume has an impact on hospital and surgeon performance in terms of costs, and quality of care [12, 13, 18, 42]. Higher prices paid to the hospitals/physicians without accompanying improvements in quality, satisfaction, or outcomes, can generate inefficiency in the system [43]. Hospital competition can also affect treatment received by patients through adoption of new procedures and technologies. In a recent study Wright et al., showed that patients in a more competitive environment are more likely to receive robotic assisted procedures compared to less competitive hospital environment [8]. Thus, understanding the process through which hospital competition affects outcomes can aid in development of appropriate policy tools.

### Limitations

We note following limitations to our study. Due to the observational data, we were not able to establish a causal relationship between hospital competition and outcomes. Patients may have changed hospitals in the long-term, which we did was not capture in our analysis. Our measure of HHI was static and did not account for changes in competition over the study period. Our data is clustered in nature, however, we focused on the association between hospitals (HHI) and outcomes at the patient level. Our analyses did not include intermediate outcomes like cancer recurrence, and change in disease severity. Additionally, there was differential length of follow-up used in the assessment of long-term mortality. We used HHI as a measure of hospital competition, and limitations of HHI are applicable. Our cohort consisted of fee-for-service Medicare beneficiaries aged ≥ 66 years and living in a SEER region. While the age and race and ethnic distribution for persons ≥ 66 years is comparable with that of older adults in the US, the SEER regions have a higher proportion of non-white persons. Mortality rates derived from SEER data may not be representative of national data on cancer mortality rates [25].

## Conclusions

Our results provide important insights regarding association between hospital competition, quality of PCa outcomes, and its variation across African American and white patients. We note several strengths of our study, including focus on fee-for-service Medicare beneficiaries, thus minimizing potential bias due to health insurance; short-term and long-term outcomes; and accounting for patient level clinical and demographic attributes. In the world of finite resources, hospital competition is an important tool for efficient resource allocation. Although healthcare is not a perfect competition environment, hospital competition is associated with improved outcomes of care, as we observed. In addition, improved hospital competition may help lower racial disparity in outcomes. Our study results have important implications for patients as we observed that higher competition is associated with improved quality of care outcomes, lower Medicare expenditure and lower racial disparity. Thus, results our study support policies that improve hospital competition such as reducing barriers to entry, implementation of antitrust laws, and minimizing anticompetitive measures. The policy implication of our study for CMS is that reimbursement policies should enhance competitive environment. This poses a significant challenge to payment reform. Future research should investigate process through which hospital competitions improves quality of care outcomes.

### Supplementary Information

**Additional file 1.**

**Additional file 2.**

## Data Availability

The data that support the findings of this study are available from SEER-Medicare. Restrictions apply to the availability of these data, which were used under license for this study. Data are available from the authors with the permission of SEER-Medicare.
