# Supplementary figures and images for: Association between hospital competition and quality of prostate cancer care
Source: BMC Health Serv Res. 2023 Aug 5;23:828. doi: 10.1186/s12913-023-09851-4 (PMC10403840; doi:10.1186/s12913-023-09851-4)

**eFigure 1: Mean Herfindahl-Hirschman Index (HHI) for hospitals, 1998-2011**

**
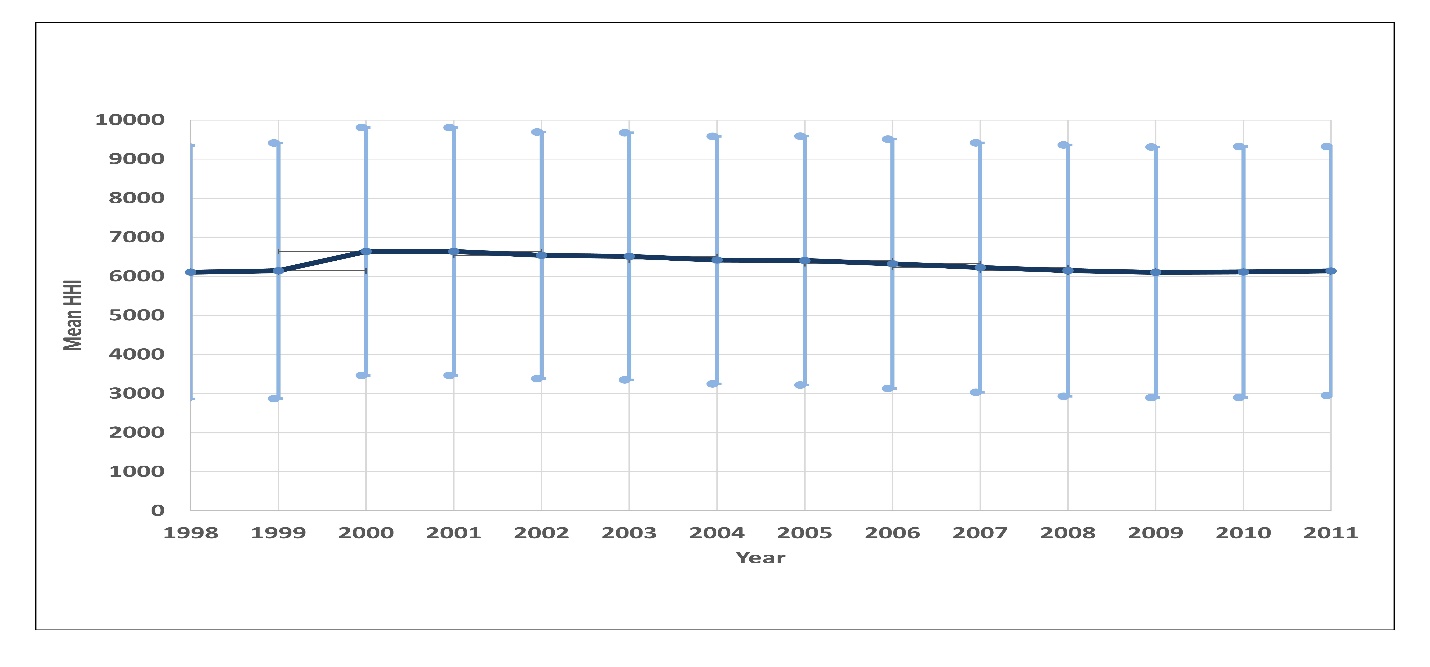
**

Supplement: Supplementary file 1 — Additional file 1. [file 12913_2023_9851_MOESM1_ESM.docx]
