# Supplementary material for: Association between hospital competition and quality of prostate cancer care
Source: BMC Health Serv Res. 2023 Aug 5;23:828. doi: 10.1186/s12913-023-09851-4 (PMC10403840; doi:10.1186/s12913-023-09851-4)
Supplement: Supplementary file 2 — Additional file 2. [file 12913_2023_9851_MOESM2_ESM.docx]

eTable 1: Diagnosis codes, procedure codes list for prostate cancer treatments

|  | **ICD 9 diagnosis code** | **ICD 9 procedure code** | **HCPCS/CPT code** |
| --- | --- | --- | --- |
| Surgery:  Radical prostatectomy , Laparoscopic (radical prostatectomy, Robotic-assisted laparoscopic radical prostatectomy) |  | 17.42, 40.1 - 40.3, , 40.5, 40.53, 40.59, 60.21, 60.29, 60.2–60.6, 60.51-60.59, 60.61, 60.62, 60.69, 62.3 | 54690, 55810, 55812, 55815, 55821, 55831, 55840, 55842, 55845, 55866, 55899, S2900 |
| Radiation | V58, V58.0,  V66.1, V67.1 | 60.99, 92.2, 92.21-92.29, 92.3, 92.30-92.39, 92.4, 92.41 | 0073T, 0082T, 0182T, 0197T, 55860, 55862, 55865, 55875, 55876, 61793, 76872, 76873, 76965, 77261–79999, C1715, C1717, C2638-C2641, C2698, C2699, G0173, G0251, G0256, G0261, G0339, G0340, G0458, G6003, G6005, G6006, G6015, Q3001, S8049  Revenue center codes 0330 or 0333, 0339 or 0342 |
| Chemo | V58.1  V66.2, V67.2 | 99.25 | 95990, 95991, 96400-96549, 96530, J0640, J2405, J8520-J8999, J9000-J9999K0415, K0416, Q0083-Q0085, Q0179, S0177, S0181  Revenue center codes 0331, 0332, or 0335  For 2005 only:G0355-G0363, G9021-G9032 |
| Hormone (androgen deprivation therapy or ADT) | V07.59 |  | C9216, C9430, G0356, G9132, J0128, J1050, J1051, J1950, J3315, J9165, J9202, J9217-J9219, S0165, S0175, S9560 |
